# A New Zinc Phosphate-Tellurite Glass for Magneto-Optical Applications

**DOI:** 10.3390/nano10091875

**Published:** 2020-09-18

**Authors:** Mihail Elisa, Raluca Constantina Stefan, Ileana Cristina Vasiliu, Stefan Marian Iordache, Ana-Maria Iordache, Bogdan Alexandru Sava, Lucica Boroica, Marius Catalin Dinca, Ana Violeta Filip, Aurelian Catalin Galca, Cristina Bartha, Nicusor Iacob, Madalin Ion Rusu, Mihai Eftimie, Victor Kuncser

**Affiliations:** 1 National Institute of R & D for Optoelectronics, INOE 2000, 409 Atomistilor Str., 077125 Magurele, Jud. Ilfov, Romania; elisa@inoe.ro (M.E.); raluca.iordanescu@inoe.ro (R.C.S.); icvasiliu@inoe.ro (I.C.V.); stefan@3nanosae.org (S.M.I.); anaducu@3nanosae.org (A.-M.I.); madalin@inoe.ro (M.I.R.); 2 National Institute for Laser, Plasma and Radiation Physics, 409 Atomistilor Str, 077125 Magurele, Jud. Ilfov, Romania; lucica.boroica@inflpr.ro (L.B.); marius.dinca@inflpr.ro (M.C.D.); ana.filip@inflpr.ro (A.V.F.); 3 National Institute of Materials Physics, Atomistilor 405A, 077125 Magurele, Jud. Ilfov, Romania; ac_galca@infim.ro (A.C.G.); cristina.bartha@infim.ro (C.B.); nicusor.iacob@infim.ro (N.I.); kuncser@infim.ro (V.K.); 4 Department Science and Engineering of Oxide Materials and Nanomaterials, University Politehnica of Bucharest, 313 Spl. Independentei, 060042 Bucharest, Romania; mihai.eftimie@upb.ro

**Keywords:** glass, diluted ferromagnetism, optical spectroscopies, magneto-optical functionalities

## Abstract

This work investigates the structural, magnetic and magneto-optical properties of a new zinc phosphate-tellurite glass belonging to the 45ZnO-10Al_2_O_3_-40P_2_O_5_-5TeO_2_ system. The glass was prepared by a wet method of processing the starting reagents followed by suitable melting–stirring–quenching–annealing steps. Specific parameters such as density, average molecular mass, molar volume, oxygen packaging density, refractive index, molar refractivity, electronic polarizability, reflection loss, optical transmission, band gap and optical basicity have been reported together with thermal, magnetic and magneto-optical characteristics. Absorption bands appear in the blue and red visible region, while over 600 nm the glass becomes more transparent. FTIR and Raman spectra evidenced phosphate-tellurite vibration modes proving the P_2_O_5_ and TeO_2_ network forming role. Magnetic measurements reveal the diamagnetic character of the Te-doped glass with an additional weak ferromagnetic signal, specific to diluted ferromagnetic oxides. Positive Faraday rotation angle with monotonous decreasing value at increasing wavelength was evidenced from magneto-optical measurements. The final product is a composite material comprising of a non-crystalline vitreous phase and Te-based nanoclusters accompanied by oxygen vacancies. The metallic-like Te colloids are responsible for the dark reddish color of the glass whereas the accompanying oxygen vacancies might be responsible for the weak ferromagnetic signal persisting up to room temperature.

## 1. Introduction

Recently, phosphate-tellurite glasses and Er^3+^-doped phosphate-tellurite glasses have been prepared and their magnetic and magneto-optical properties have been carefully investigated [1]. The uptake of Er^3+^ ions into the glass increases the value of the paramagnetic susceptibility. The results confirm the improvement of magneto-optical properties of the examined glasses under the effect of doping with erbium ions, the reported values of the Faraday rotation angles being comparable with the ones observed in lanthanum-type tellurium glasses [2].

Optical current transducers based on magneto-optical tellurite glass fibers were also recently reported [3,4] whereas other tellurite glasses doped by rare-earth ions have been at the latest investigated in relation to their magneto-optical properties as well [4,5,6].

Sodium zinc tellurite sol–gel glass films were explored in relation with the mechanism of the TeO_2_ reduction to metallic-Te [7]. It was demonstrated that the formation of metallic-Te is responsible for the decrease of the optical transmission in the Vis domain. In order to minimize the TeO_2_ reduction process, oxygen gas purge was applied accompanied by the heat treatment of the films at 300–400 °C, which resulted in a bleaching of the glass films and increasing of the visible transmission.

Bismuth zinc tellurite Radio Frequency (RF) magnetron reactive sputtered glass films were synthesized aiming to improve the optical transmission in the visible range [8]. It was demonstrated that the O_2_ purge into Ar working gas improved the optical transparence of the films in the visible domain by suppressing the reduction process of TeO_2_ into metallic-Te atoms.

It is worth mentioning the previous results of the authors related to magnetic and magneto-optical properties of Dy^3+^/Tb^3+^/La^3+^ alumina-phosphate vitreous systems applied as bulk Faraday rotators [9,10,11] as well as to the rare-earth-doped silico-phosphate sol–gel thin films for magneto-optical applications [12]. A very recent work of the authors also referred to thermal, structural, mechanical, magnetic and magneto-optical properties of Bi_2_O_3_ and PbO-doped boron-phosphate glasses [13].

The present work is focused on the investigation of optical, structural, magnetic and magneto-optical properties of a newly prepared zinc phosphate-tellurite glass doped with TeO_2_, having encouraging interesting magnetic properties and magneto-optical functionalities, being, therefore, of fundamental research interest but also a good candidate to be used as a Faraday rotator over 600 nm wavelength. Some physicochemical properties have been measured and calculated such as density, refractive index, characteristic temperatures, molar volume, oxygen packaging density, molar refractivity, polarizability, optical band gap, optical basicity and metallization criteria, being compared with recently investigated tellurite glasses [14,15,16,17,18]. The gain of the work is related to the application of the non-conventional wet method of chemical precursors processing in phosphoric acid solution that ensures a high chemical and optical homogeneity of the final glass. The reported investigations give support for a new zinc phosphate-tellurite glass composition having an improved chemical steadiness, a reduced crystallization tendency together with magnetic and magneto-optical properties, which have been also extensively investigated. On our knowledge, this oxide glass composition has not been investigated so far. The work emphasizes the correlation between metallic-Te_2_ nanoclusters accompanied by oxygen vacancies giving rise to nanoclusters of magnetic defects, which can be either isolated or in magnetic interaction, providing the weak ferromagnetic signal, which exists up to room temperature. This confers to the reported oxide glass composition based on only diamagnetic elements, unique magnetic and magneto-optic properties specific to a dilute magnetic oxide nanocomposite system, which can be easily actuated by reasonable low magnetic fields. The study of this novel phosphate-tellurite glass for Faraday rotators is motivated by the interest in approaching a new composition and the correlation between its optical, structural, magnetic and magneto-optical properties.

## 2. Materials and Methods

### 2.1. Preparation of Glass

The vitreous material prepared in this work belongs to the 45ZnO-10Al_2_O_3_-40P_2_O_5_-5TeO_2_ system (code Te-5, taking into account the amount of TeO_2_ in mol. %), being characterized by a chemical stable composition. The glass has been prepared by a non-conventional wet method of processing the starting reagents (high purity ZnO, TeO_2_, Al_2_O_3_ and H_3_PO_4_ solution, density 1.71 g/L, 80 wt. % concentration, purchased from standard producer Sigma-Aldrich, St. Louis, MO, USA), followed by melting–stirring and annealing of the glass. The glass polishing was done in order to prepare appropriate samples for specific characterization techniques. In order to prepare a glass having a high optical homogeneity, the melted batch was mechanically stirred aiming to reduce the gaseous inclusions and grooves embedded in the bulk samples. The glass preparation steps are the following: i) starting glass batch was prepared in a quartz bowl by dissolving the precursor reagents in H_3_PO_4_ solution followed by stirring and evaporation of the raw materials (chemical reagents) at 180 °C, for 6 h on an electrical hob; ii) preliminary heat treatment of the glass batch at 700 °C, for 7.5 h, using an alumina crucible; iii) melting and stirring of the glass batch at 1100 °C, for 30 min, using the same alumina crucible; iv) casting of the glass melt in preheated graphite molds; v) annealing of the glass at 390 °C (below the glass transition temperature, *T_g_*), for 30 min, in order to release the remnant stress aroused during the molding stage; vi) polishing of the glass using SiC and CeO_2_ powders. The benefits of the wet non-conventional technique were presented in a recent work of the authors related to magneto-optical properties of Bi_2_O_3_ and PbO-doped aluminophosphate glasses [19] valid also for phosphate-tellurite glass from the present study.

### 2.2. Measurements

The X-ray diffraction (XRD) measurement was performed at room temperature, in the 10–80° range, with a 0.05° step and 3 s integration time, using a Bruker D8 Advance device (CuKα, λ = 1.54056 Å), Billerica, MA, USA. The analysis of the thermal properties was performed on a SETARAM SETSYS Evolution 18 instrument, Caluire, France, in a Thermogavimetry-Differential Scanning Calorimetry TG–DSC Thermal Analyzer mode, using an open cylindrical alumina crucible. The measurements have been performed in an inert atmosphere of Ar, from room temperature (RT) up to 1000 °C, using a heating rate of 10 °C/min, with a 16 mL/min gas flow rate. The accuracy of heat flow measurements was ± 0.001 mW and the temperature precision of ± 0.1 K.

The thermal expansion coefficient was determined by the dilatometry/dilatometric method with the horizontal Netzsch DIL 400 PC device, NETZSCH Holding, Selb, Germany. The temperature was increased at 3 °C/min. The characteristic temperatures (strain point, *T_S_*, transition temperature, *T_g_*, annealing point, *T_A_,* and softening point, *T_D_*) as well as the thermal expansion coefficient ($\alpha_{20}^{300}$) of the glass were estimated by processing the results from the thermal analysis. The measurement errors for the thermal expansion coefficient were in the range ± 0.8·10^−7^ K^−1^, provided by calibration with the SiO_2_ standard sample, according to ISO 7991.

The density of the glass was experimentally determined by the hydrostatic method and the refractive index was measured at *λ_D_* = 589 nm (yellow doubled *D* line of sodium) by means of a Pulfrich refractometer.

The optical and magneto-optical measurements (conventional spectroscopy and transmission ellipsometry) were carried out with a Woollam Variable Angle Spectroscopic Ellipsometer, VASE (J.A. Woollam Co., Lincoln, NE, USA), equipped with a high-pressure Xenon discharge lamp (Hamamatsu Photonics K.K., Japan), incorporated in an HS-190 monochromator (J.A. Woollam Co., Lincoln, NE, USA). The samples (rectangular pieces of optical quality polished glass with precisely determined thickness) have been aligned perpendicularly to the incident light beam. The magnetic field-assisted Faraday rotation measurements were performed with the aid of a toroidal permanent magnet, and the field applied parallel to the wave-vector.

Fourier transform infrared (FTIR) spectroscopic measurements were carried out at RT, in the range 500–1500 cm^−1^, using a Perkin Elmer Spectrophotometer-Spectrum 100 provided with Universal Attenuated Total Reflectance (UATR) accessory Llantrisant, UK, in transmission and universal attenuated total reflection (ATR, ZnSe/Germanium window) modes. Raman spectra were collected at RT with a LABRAM-HR 800 Horiba Jobin Yvon spectrometer, (Montpellier, France), in the 200–1500 cm^−1^ range, by using Ar^+^ laser excitation (*λ* = 514.5 nm) with the laser power at the sample surface of 5 mW, resolution of 0.5 cm^−1^, laser spot diameter of 1 μm and grating of 1800 L/mm.

The magnetic behavior of the sample was investigated over a large range of applied magnetic fields and temperatures by using a superconducting quantum interference device (SQUID) magnetometer (MPMS 7T from Quantum Design, CA, USA).

## 3. Results and Discussion

### 3.1. Physicochemical Properties

**Figure 1:**
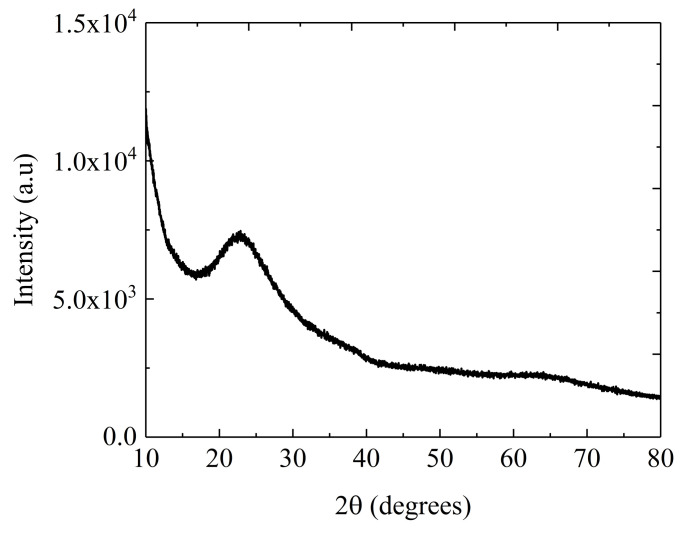
XRD pattern of Te-5 glass.

The X-ray diffraction pattern acquired at room temperature and presented in Figure 1, confirms the amorphous state of the investigated material.

Some important physicochemical properties of Te-5 glass are presented in Table 1, such as density (*ρ_glass_*), average molecular mass (*M_av_*), molar volume (*V_M_*), oxygen packing density (*OPD*), refractive index measured at 589 nm (*n_D_*), refractive index measured from the dispersion graph (*n_DD_)*, molar refractivity (*R_m_*), electronic polarizability (*α_m_*), reflection loss (*R_L_*) and optical transmission (*T*).

The density of glass, *ρ_glass_*, is an important physical property that estimates the compactness of the structure of a glassy system. It depends on the glass composition and it is mainly influenced by the glass network structure, preparation conditions and thermal history. The higher is the average molecular mass, *M_av_,* that is, the higher the molecular weights of the components are, the higher the density of the glass is. The density of Te-5 glass is lower by comparison with other high TeO_2_ content glasses (over 60 mol. %) [14,16,18].

In the case of the vitreous systems, the molar volume, *V_M_*, reveals the network structure and the arrangement of the component units as it is directly correlated with the 3D structure/disposal of the oxygen atoms. Generally, the molar volume of the glasses increases due to the increase of the bond length or interatomic arrangement of metal ions/anions. Due to a lower density, the molar volume of the Te-5 glass is higher than that of recently investigated high TeO_2_ content glasses [15,16,17,18], showing a less compact vitreous network.

Oxygen packing density, *OPD*, may be associated with the number of oxygen atoms in a glass chemical composition unit (*C*) and a glass volume unit. It is calculated based on the equation [15,17]:

(1) $$OPD=\frac{C}{V_{M}}$$

where *C* represents the number of oxygen atoms per glass mole and *V_M_* is the molar volume. Thus, the oxide glass composition and the molar volume are decisive for the *OPD* value. In the case of Te-5 glass, *OPD* was relatively close to the values characteristic for the high TeO_2_ content glasses [15,17].

The refractive index of glass, *n_D_,* was influenced by the interaction of light with the electrons of the component atoms of the glass. The refractive index is an important feature of the optical glasses being closely connected with the electronic polarization of the ions and the local field inside the glass, mainly in connection with the electronic structure of the glasses. The non-bridging oxygen atoms are connected by cations (vitreous network modifiers) through ionic bonds whereas bridging oxygen atoms are connected by two phosphorous atoms (vitreous network formers) through covalent bonds. The ionic bonding results in a high polarizability of the non-bridging oxygen atoms (deformation of the outer electron shell in ligand fields) as compared to covalent bonding of the bridging oxygen atoms. Consequently, the decrease in the number of the non-bridging oxygen atoms determines the decrease of the refractive index value. In the present study, Te-5 glass, due to a low TeO_2_ content, exhibits a refractive index value that is considerably lower than that of other high TeO_2_ content glasses [14,15,16,17]. This can be explained by a decreased number of high polarizability Te atoms in the explored glass.

The dielectric constant (relative permeability), *ε*, is a parameter that characterizes the electrical polarization state of the glass and is calculated using the following equation, under the implicit assumption of a relative magnetic permeability of 1 [15,16]:

(2) $$\varepsilon =n^{2}$$

In the case of Te-5 glass, *ε* = 2.39, being lower than that of other high TeO_2_ content glasses [15,16]. This is due to a reduced number of high polarizability Te atoms from the nanoclusters, these ones being responsible for the relative reduced electrical insulator character of the glass.

The molar refractivity (refraction) is the extent of the total polarizability per unit mole of glass. The Lorentz–Lorenz equation is used to correlate the molar refractivity, *R_m_*, with the refraction index, *n,* and molar volume, *V_M_* [14,15,16,17]. Actually, the molar refractivity provides the average molar refraction for substances that are isotropic in nature, such as glasses. The molar refractivity can also be calculated with dependence on the electronic polarizability, *α_m_,* of a molecule, which measures the suitability of electrons to respond to an electric field [14,15]. The non-bridging oxygen atoms have a high tendency to polarize compared to bridging oxygen atoms. So, the higher the number of non-bridging oxygen atoms in the glass network is, the higher the molar refraction and electronic polarizability are. In the case of Te-5 glass, the molar refractivity and electronic polarizability have about the same values by comparison with the glasses containing about 60 mol. % TeO_2_ [16,17] but lower values by comparison with other tellurite glasses containing more than 70 mol. % TeO_2_ [14,15]. Thus, the investigated glass tends to be less polarized when an electric field is applied, comparatively to glasses containing more than 70% TeO_2_.

Reflection loss, *R_L_*, and optical transmission, *T*, were calculated based on the refractive index, *n_D_,* and for the Te-5 glass samples, *R_L_* and optical transmission, *T* show lower and higher values, respectively, by comparison with recently investigated tellurite glasses [15,16].

### 3.2. Optical Properties and Related Electron Structure Parameters

**Figure 2:**
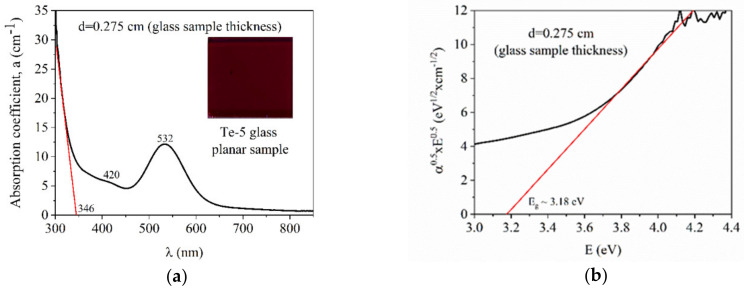
(**a**) The optical absorption of the Te-5 glass sample (inset-planar glass sample image) and (**b**) the graphical determination of the energy band gap, *E_g_*.

The absorption spectrum of Te-5 glass in the UV-Vis-NIR domain is presented in Figure 2a. Two absorption bands at 420 and 532 nm were observed, specific to the electronic transitions of the Te_2_ diatomic molecules that were formed as a result of TeO_2_ decomposition at the melting temperature.

At 1100 °C, the decomposition of TeO_2_ in metallic Te atoms takes place with the enhanced probability of giving rise to oxygen vacancies in the glass. As a result, Te atoms link to each other, resulting in Te_2_ diatomic molecules that are responsible for absorption and luminescence characteristics. The absorption band from 432 nm is assigned to the ^3^Σ_g_ → ^3^Σ_u_ transition whereas the strong 532 nm absorption band is due to colloidal metallic Te [15,16,17,18,19,20,21,22]. Thus, according to these data, the final product is a composite material comprising of a non-crystalline vitreous phase and Te_2_ nanoclusters, the latter giving the dark reddish color of Te-5 glass (see inset from Figure 2a). Taking into consideration that diatomic tellurium molecule-based nanoclusters exhibit a green absorption range (see Figure 2a), the complementary transmitted light is noticed in the red domain of the visible spectrum.

In Figure 2b, the graphical determination of *E_g_* for Te-5 glass sample is presented [14,15,16,17]. The Mott and Davis/Tauc equation was applied to graphically determine the optical band gap, *E_g_* (Figure 2b). Thus, the equation [14,15,16,17]:

(3) $$\alpha h\nu ={\left(h\nu -E_{g}\right)}^{n}$$

allows one to determine the band gap, *E_g_*, where *α* is the absorption coefficient, *h* is the Planck constant, *ν* is the light frequency and *n* can be ½ for the allowed direct electron transitions and 2 for the allowed indirect electron transitions from the valence to the conduction band [23]. For amorphous materials, in the case of Te-5 glass, *n* takes the value 2. The optical band gap, *E_g_* value, was obtained by extrapolating the linear region of the curve to the zero absorption at which, αhν=0, and the result was *E_g_*_=_ 3.18 eV (see Table 2). The wavelength corresponding to the band gap value was *λ_g_* = 390 nm, which was different from the cut-off wavelength deduced from Figure 2a, which was *λ_cutt-off_* = 346 nm.

**Figure 3:**
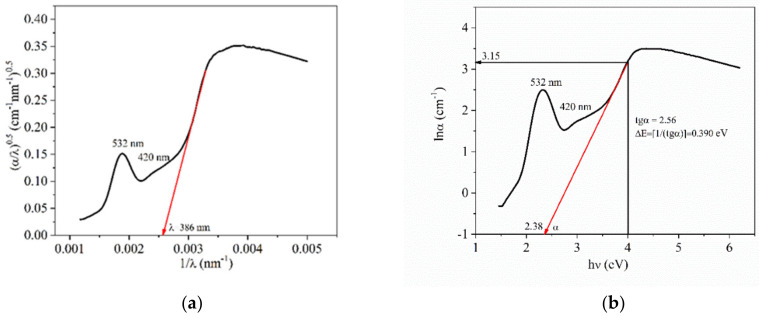
(**a**) Variation of (α/λ)^0.5^ with *1/λ*; and(**b**) Urbach plot for Te-5 glass.

In order to verify the validity of the graphical method to determine *E_g_* and, respectively, *λ_g_*, the absorption spectrum fitting (ASF) method was applied [15,16,17] by means of which, $E_{opt}^{ASF}$ was determined and it was compared to the *E_g_* value determined by Mott and Davis/Tauc law_._ Thus, the Tauc law is written as Equation (4) [15,16,17]:

(4) $$\frac{\alpha hc}{\lambda }={\left(\frac{hc}{\lambda }-E_{g}\right)}^{2}$$

where, *α* is the absorption coefficient, *h* is the Planck constant and *c* is the light speed in vacuum equal to 3 × 10^8^ m/s, *λ* is the wavelength and *E_g_* is the band gap energy_._ Further on, it is graphically represented as ${\left(\alpha /\lambda \right)}^{0.5}=f\left(\frac{1}{\lambda }\right)$ (Figure 3a). The result is a curve and the tangent to the linear region of the curve will intersect the *x*-axis in a point representing *1/λ_g(ASF)_*, resulting in *λ_g(ASF)_*. This value is compared to that determined from Tauc law. In our case, *1/λ_g(ASF)_* = 0.00259 and *λ_g(ASF)_* = 386 nm, which is relatively close to that determined from Tauc law, *λ_g_* = 390 nm. The Equation (5) [15,16,17]:

(5) $$E_{opt}^{ASF}=\frac{1240}{\lambda_{g}}$$

is applied to calculate $E_{opt}^{ASF}$, which, in our case is3.21 eV and is relatively close to 3.18 eV that is graphically determined by the Tauc law.

Generally, for amorphous materials, the allowed indirect electron transitions according to the Tauc equation are valid. In the case of optical absorption, for low photon energy ranged between 10^2^ and 10^4^ cm^−1^, the absorption coefficient follows Urbach’s law. This is the width of the band tails of the localized states from the valence band. Urbach’s law is depicted by the following relationship (6) [14,16,24]:

(6) $$\alpha \left(\vartheta \right)=\alpha_{0}\exp\left(\frac{h\vartheta }{\Delta E}\right)$$

where, *α(ν)* is the absorption coefficient dependent on the frequency, *hν* is the energy and Δ*E* is the Urbach energy. If the logarithm of Urbach’s relationship is applied, the following Equation (7) is obtained [14,16,24]:

(7) $$ln\alpha \left(\vartheta \right)=C+\frac{h\vartheta }{\Delta E}$$

Hence, it is possible to deduce the Δ*E* value.

In the case of Te-5 glass, Urbach energy, Δ*E,* was 0.390 eV (Figure 3b). Urbach energy is a measure of the disorder degree of the vitreous materials and is correlated with an extension of the localized states within the band gap. Accordingly, the disorder degree of the investigated glass is comparable with that of high TeO_2_- containing glasses [14,16].

**Figure 4:**
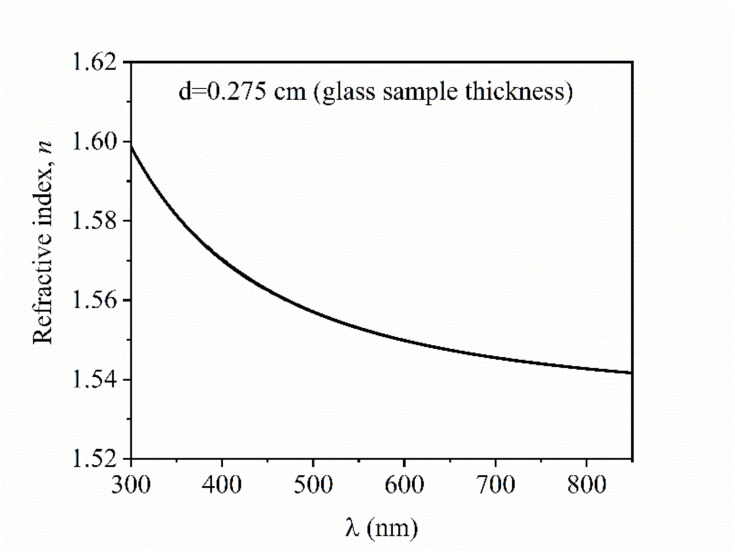
The refractive index versus wavelength for the Te-5 glass.

The refractive index dependence on wavelength (optical dispersion) is presented in Figure 4. It can be observed that the refractive index value measured at 589 nm (*n_D_*, the yellow line of sodium) was about 1.550, close to 1.546, measured value by the Pulfrich refractometer, thus confirming the accuracy of those two measurement methods.

One should note that the measurement modes of the refractive index and optical absorption coefficient are different. The optical absorption is derived from conventional transmission data, in this case, the probing light interacting with the sample volume. On the other hand, the refractive index is inferred from the ellipsometry data, which, in this case, is giving information only from the sample surface and the raw data do not contain signatures of the absorption bands discussed above. Therefore, the presented optical properties are not Kramers–Kronig correlated.

Table 2, besides the *E_g_* value that has already been discussed, presents other optical properties of Te-5 glass such as refractive index-based oxide ion polarizability, $\alpha_{O_{2}^{-}}\left(n\right),\;$ band gap-based oxide ion polarizability, $\alpha_{O_{2}^{-}}\left(E_{g}\right),\;$ refractive index-based metallization criterion, *M(n)*, band gap-based-metallization criterion, *M(E_g_)*, Duffy and Ingram theoretical optical basicity, *Λ*_th_, refractive index-based polarizability-based optical basicity, *Λ(*$\alpha_{O_{2}^{-}}\left(n\right)$, band gap-based polarizability-based optical basicity, Λ($\alpha_{O_{2}^{-}}\left(E_{g}\right),$ and Pauling optical basicity, Λ_P_ [14,15,16,17].

Dimitrov and Komatsu calculated the oxide ion polarizability, $\alpha_{O_{2}^{-}}$, optical basicity, *Λ,* and metallization criterion, *M*, on the basis of two different properties: energy band gap, *E_g_,* and linear refractive index, *n*. Thus, the calculation of the refractive index-based oxide ion polarizability, $\alpha_{O_{2}^{-}}\left(n\right),$ is based on the molar refractivity, *R_m_*, total electronic polarizability of the compounds from the glass, $\sum \alpha_{i},$ and the number of oxygen atoms from each oxide, $N_{O_{2}^{-}}$, reported in [14,17].

The estimation of the energy band gap-based oxide ion polarizability, $\alpha_{O_{2}^{-}}\left(E_{g}\right),$ is based on the molar volume, *V_M_*, band gap, *E_g_*, the total polarizability of cations, $\sum \alpha_{i},$ and the number of oxygen atoms in the molar oxide glass composition, $N_{O_{2}^{-}}$, reported in [14,17]. In the case of Te-5 glass, $\sum \alpha_{i}=0.234;$ $N_{O_{2}^{-}}=2.85$, and, based on the measured *n_D_* value (see Table 1), it was found $\;\alpha_{O_{2}^{-}}\left(E_{g}\right)=3.095\;{Å}^{3}.$ Refractive index-based oxide ion polarizability and energy band gap-based oxide ion polarizability values, in the case of Te-5 glass, are lower by comparison with high content TeO_2_ glasses [12,15] (see Table 2), due to the low tellurium oxide content from the vitreous material.

According to the metallization theory in condensed matter by Herzfeld [25], the metallization parameter, *M*, is theoretically calculated to estimate the tendency for metallization and to investigate the insulating behavior of a sample. This theory infers that the refractive index becomes infinite under the condition that *R_m_/V_M_ = 1*, which is correlated with a covalent bonding. This ratio is related to the degree of electron localization, showing the tendency of a material to become metallic in nature [14,15,16]. The case of *R_m_/V_M_ > 1* means a metallic nature (delocalized electrons) whereas *R_m_/V_M_ < 1* means a non-metallic nature (localized electrons). The large value of the metallization parameter *M*, defined as *1- R_m_/V_M_* demonstrates a high energy band gap and insulating nature in the synthesized glasses, which is confirmed by the optical band gap energy, *E_g_*. A larger *M* value indicates that the widths of both valence and conduction bands decrease, which results in a wider band gap [16,26].

Metallization criterion, *M*, is calculated based on the relationship that uses *R_m_* and *V_m_* and also based on the refractive index, *n* and optical band gap, *E_g_*_,_ respectively [14,15,16]. In the case of Te-5 glass, metallization criterion, *M*(*R_m_/V_m_*) = *M(n*) = 0.683 and *M(E_g_)* = 0.398, demonstrating a relative significant insulator behavior of the vitreous material, by comparison with high TeO_2_ content glasses, which demonstrated a more conductive character [14,15,16].

The understanding of glass optical basicity could be beneficial for the fabrication of novel useful materials with better optical performances. The degree of acidity or basicity of glass is related to the electron donor power of oxygen atom and it is influenced especially by the glass network formers. High optical basicity means high electron donor ability of the oxide ions to the cations [16]. The optical basicity, *Λ*, that is strongly influenced by the electronic polarizability, *α_m_*, has been evidenced to be a determinant and a principal parameter for predicting the properties of a glass system before using the glass in various applications [27].

The relationship proposed by Duffy and Ingram for the theoretical value of optical basicity of multi-component glasses is applied to obtain energy band gap based-optical basicity and refractive index based-optical basicity. Duffy and Ingram proposed a relationship to determine the theoretical optical basicity, *Λ_th_*, for multi-component glasses based on the optical basicity of each oxide component multiplied by the equivalent fraction of each component [14]. Duffy has established an alternative approach of refractive index-based polarizability-based optical basicity, Λ($\alpha_{O_{2}^{-}}\left(n\right),$ and band gap-based polarizability-based optical basicity, *Λ(*$\alpha_{O_{2}^{-}}\left(E_{g}\right)$ [14]. Other equations are used to determine optical basicity based on elemental Pauling electronegativity [16,17]. In the case of Te-5 glass, *Λ_th_* = 0.702, *Λ(*$\alpha_{O_{2}^{-}}\left(n\right)$ *= Λ(*$\alpha_{O_{2}^{-}}\left(E_{g}\right)$ = 1.13 and Λ_P_ = 0.4617. It is evident that the theoretical optical basicity value in the case of the Te-5 glass was close to the basicity calculated based on the oxide polarizability dependent on the refractive index and, respectively, on the energy band gap. It means that, in our case, the calculation models proposed by Duffy and Ingram and, respectively, Duffy, are valid and match Te-5 glass composition. Taking into consideration the optical basicity values, it is worth claiming that the Te-5 glass has an intermediate basicity value. Glass optical basicity is related to the electron donor power of oxygen atoms. The higher electron donor power of oxygen atoms means a reduced degree of covalence cation-oxygen that is an increase in the ionic character of the bond resulting in a reduced tendency to form the vitreous structure. The basicity of Te-5 glass is mainly influenced by ZnO (amphoteric character) and P_2_O_5_ (acidic character), both of them being in a high amount by comparison with Al_2_O_3_ and TeO_2_. It can be concluded that the present glass composition has an intermediate tendency to form the vitreous network.

### 3.3. Thermal Properties

**Figure 5:**
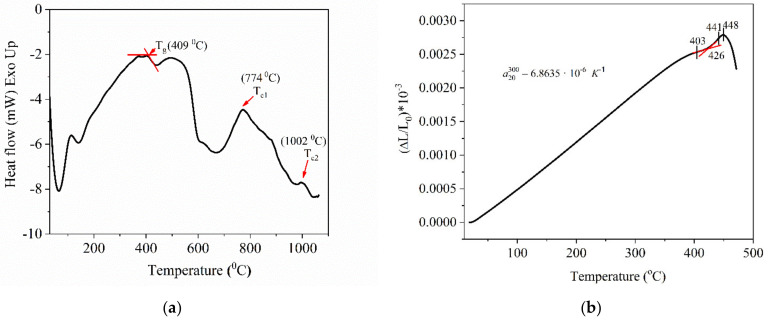
(**a**) Differential Scanning Calorimetry (DSC) thermal scan of the Te-5 glass sample and (**b**) the dilatometric thermal expansion plot of the Te-5 glass.

The DSC curve shown in (Figure 5a) was obtained by subtracting the background corresponding to the alumina crucibles from the measured data. The obtained DSC signal corresponded to the analyzed sample. The glass transition temperature (*T_g_*) as determined from the DSC data is about 409 °C (Figure 5a). It is noticed from the DSC curve (Figure 5a), the appearance of two exothermic peaks, at 774 °C (*T_c1_*) and 1002 °C (*T_c2_*), corresponding to different possible devitrification phenomena, providing phosphate and tellurite compounds [28]. The crystallization mechanisms will be investigated in a separate work.

In Figure 5b, the dilatometric thermal expansion plot is presented, from which, the thermal expansion coefficient was calculated, being $\;\alpha_{20}^{300}=6.8635\cdot 10^{-6}\;K^{-1}$ and the characteristic temperatures were: strain point (*Ts*), 403 °C, transition temperature (*T_g_*), 426 °C, annealing point (*T_A_*), 441 °C, and dilatometric softening point (*T_D_*), 448 °C.

According to these values, it is worth claiming that the investigated glass has relatively high energy bonds due to a rigid phosphate-tellurite structure formed by chains and tridimensional units. There is a difference related to the glass transition temperature values determined by DSC analysis and the dilatometry method, explainable by the change of the technical details (the state of the solid sample as powder or bulk rod, the heating rate, the working atmosphere, etc.).

### 3.4. Structural Analysis

**Figure 6:**
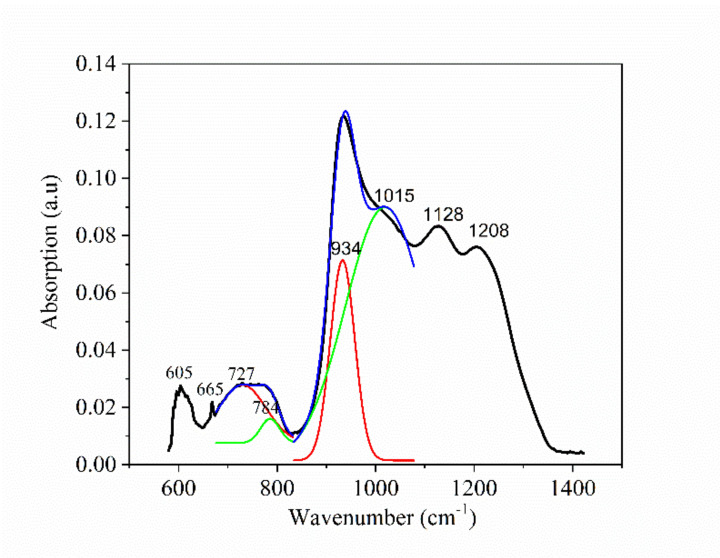
FTIR spectrum of the Te-5 glass sample.

In Figure 6, the FTIR absorption spectrum of Te-5 glass is presented in the range 600–1400 cm^−1^. Absorption peaks are noticed, being specific to Te-O and P-O bonds from the phosphate network. Thus, the absorption maximum at 605 cm^−1^ was assigned to the Te-O symmetrical stretching vibration mode from TeO_4_, overlapping the P-O-P (bridging oxygen atoms, BOs) bending vibration mode [16,17,29,30,31,32,33,34]. The band from 665 cm^−1^ is assigned to Te-O symmetrical stretching from TeO_3_/TeO_4_ [16,17,30] and P-O-P symmetrical stretching for long chains in Q^2^ units [31,32,33]. The peaks found at 727 cm^−1^ and 784 cm^−1^, resulted by the deconvolution of a large envelope, are assigned to P-O-P symmetric stretching mode in between Q^1^ and Q^2^ units and, respectively, to the P-O-P symmetric stretching mode in Q^1^ units [31,32,33,34]/Te-O symmetrical stretching vibration mode from TeO_3_ [16].

The peak from 934 cm^−1^ is allotted to the P-O-P asymmetrical vibration mode [29,32,33]/symmetric stretching of O-P-O units, NBOs (non-bridging oxygen atoms) in Q^0^ tetrahedra [30] and/or to the symmetrical stretching mode of (PO_4_)^3−^ [31,34]. The peak from 1015 cm^−1^ is assigned to the (PO_3_)^−^ asymmetrical stretching mode in Q^1^ tetrahedra [29,30]/(PO_4_)^3−^ asymmetrical stretching mode [32,33,34] and/or to symmetrical stretching mode of (P_2_O_7_)_4_^−^ [31], the maximum at 1128 cm^−1^ is ascribed to the O-P-O symmetrical stretching mode [30,31,32,33,34] and that from 1208 cm^−1^ is allotted to the O-P-O asymmetrical stretching mode in Q^2^ tetrahedra [29,31,32,33,34]. ZnO shows an absorption band from ZnO_4_ at 300 cm^−1^ that does not appear in the spectrum, being placed out of the spectrophotometer measurement range [16].

**Figure 7:**
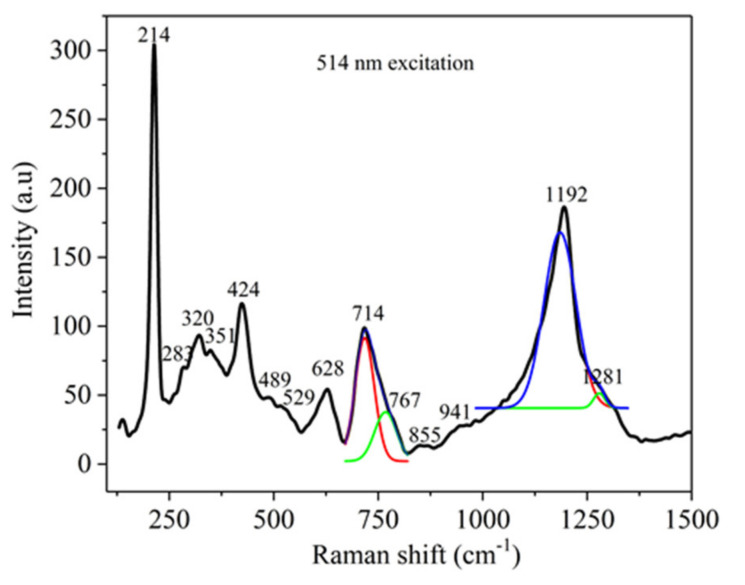
Raman spectrum of the Te-5 glass sample.

Figure 7 displays the Raman spectrum of the Te-5 glass in the range 125-1500 cm^−1^, recorded by 514 nm laser excitation [18]. It is worth mentioning the specific peak at 214 cm^−1^ assigned to the vibration modes of Te_2_ -based nanoclusters [35].

The bands that appeared at 283 cm^−1^, 320 cm^−1^ and 529 cm^−1^ are attributed to Te-O-Te bending and symmetrical stretching modes from TeO_4_, TeO_3+1_ and TeO_3_ polyhedral [16,17,29], (P_2_O_7_)^4−^ [30], the P-O-P bending vibration mode [32,33,34], the bending O-P-O vibration mode [34] and the Zn-O vibration mode from ZnO_4_ tetrahedra [16]. The peaks from 351 cm^−1^, 424 cm^−1^ and 489 cm^−1^ are ascribed to symmetric bending vibration of TeO_4_ units [16], stretching and bending of Te-O-Te bonds in TeO_4_, TeO_3+1_ and TeO_3_ units [16,17], Zn-O from ZnO_4_ units [16], harmonics of the P-O-P bending vibrations mode [33,34] and the O-P-O bending vibration mode [34], respectively. The band observed at 628 cm^−1^ is allotted to the vibration modes of the TeO_4_ continuous network [16,29] and the TeO_3+1_, TeO_3_ [17] and P-O-P bending vibration mode [34]. The maxima from 714 cm^−1^ and 751 cm^−1^ are due to symmetrical stretching of P-O-P groups in Q^2^ and Q^1^ structural units, respectively [29,30,31,32,33,34], overlapping the stretching vibration mode of non-bridged Te-O in TeO_3_ and TeO_3+1_ [16,17,29]. The peak from 855 cm^−1^ is assigned to the stretching vibration modes of TeO_3_ [16,17,29]. The peak from 941 cm^−1^ is ascribed to the P-O-P asymmetric stretching vibration mode [34]. Finally, two superposed spectral components are assigned as follows: (i) the most intense one at 1192 cm^−1^ corresponds to the asymmetric stretching vibrations of PO_2_ groups (Q^2^ structural units) [31], overlapping the (PO_3_)^2−^ asymmetrical vibration mode [32,33] and to the symmetrical stretching mode of TeO_3_ [16] and (ii) the one at 1281cm^−1^ corresponds to the asymmetrical stretching modes of PO_2_ groups in Q^2^ phosphate tetrahedral [30,31] and to the possible P=O stretching vibration mode for long phosphate chains [32,33].

### 3.5. Magnetic and Magneto-Optical Properties

**Figure 8:**
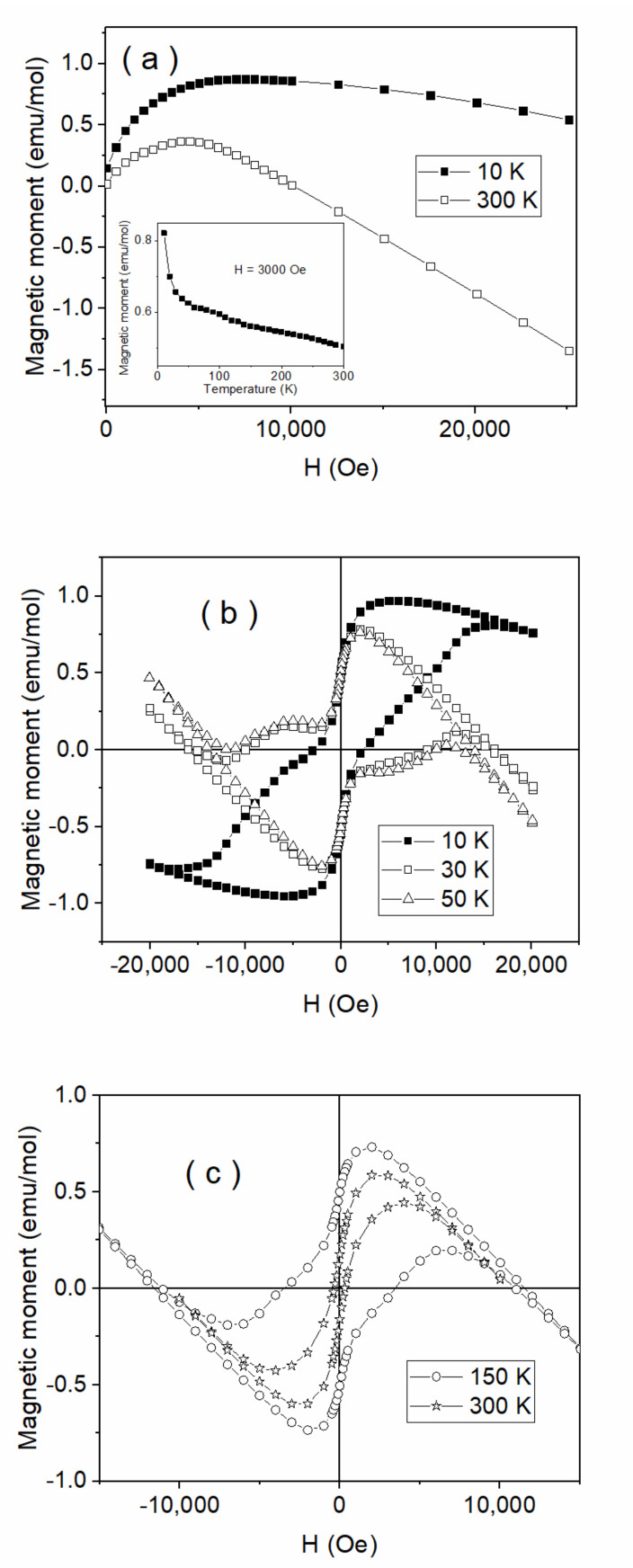
Magnetization data vs. the applied field at 2 different temperatures (**a**) with the inset presenting the dependence of magnetization vs. temperature in a field of 3,000 Oe and hysteresis loops at 10 K, 30 K and 50 K (**b**) and at higher temperatures up to 300 K (**c**).

The dependence of the magnetization versus the applied field at two different temperatures is shown in Figure 8a. It is observed in both cases that after an initial increment of the magnetization at lower fields of up to about 5000 Oe, the specific diamagnetic behavior consisting of the linear decrease of the magnetization was evidenced at higher fields. The negative slope was clearly lower in absolute value at 10 K, giving an indirect indication for an additional paramagnetic contribution of positive slope, which compensates partially for the decrease of magnetization at this temperature. The direct indication for the paramagnetic contribution can be observed from the strong increase of the magnetization at temperatures lower than 20 K (see inset of Figure 8a) resembling closely to the typical paramagnetic *C/T* type dependence with *C* the Curie constant and *T* the temperature [36]. The diamagnetic susceptibility of the Te-5 glass can be calculated from the slope of the linear decrease of the magnetization at 300 K, where the paramagnetic contribution was negligible. A value of −72(2) × 10^−6^ emu/(mol·Oe) = −81(2) × 10^−10^ m^3^/kg of the diamagnetic susceptibility *Χ_dia_* was calculated from the slope of the linear decrease of magnetization at 300 K in Figure 8a. Considering also the slope of the linear decrease of the magnetization at 10 K of only -0.22(2) × 10^−6^ emu/(mol·Oe) from the same figure, a paramagnetic contribution *Χ_para_* = +50(2) × 10^−6^ emu/(mol·Oe) = +56(2) × 10^−10^ m^3^/kg was straightforwardly deduced. Accordingly, the equation *Χ_para_* = *C/T* with *T*=10 K provides a value of 5.0(2) × 10^−4^ (emu·K)/(mol·Oe) for the Curie constant. The later one can be expressed as *C* = N_A_*μ^2^/3k_B_, where *k_B_* is the Boltzmann constant, *μ* is the magnetic moment of the magnetic center and *N_A_* is the Avogadro number [36]. A value of 2.6(1) × 10^−20^ emu or equivalently of 2.7 µ_B_ *(µ_B_* is the Bohr magneton) was obtained for the paramagnetic moment per each glass molecule (formula unit). On the other hand, the initial increase of the magnetization at lower fields, with a much higher positive slope than the paramagnetic one, had to be related to a ferromagnetic signal, which might be fully investigated via the hysteresis loops taken at various temperatures. Indeed, by cycling the magnetic field, the magnetization always opens finite loops, even at 300 K, as observed from Figure 8b,c. Such ferromagnetic signals persisting up to room temperature in materials that do not contain magnetic elements (as the present glass is) has to be related to the so called diluted magnetic oxide behavior being presently related mainly to oxygen vacancies [36,37,38]. One of the most reliable models for the long-range ferromagnetism in such oxides is the model of the magnetic polarons, which consists of compensating electronic charge distributed over nm range volumes around a specific type of oxygen vacancies [37]. While the magnetic centers can be always related to the oxygen defects around transition metals, only a part of such magnetic centers remain in the exchange interaction as overlapping polarons, leading to the long-range ferromagnetic signal. The rest of the non-interacting magnetic centers gave rise to the paramagnetic signal, which had significant contribution to only the magnetization (and not to the coercivity) only at lower temperatures. The paramagnetic contribution superposed over the diamagnetic one is the already discussed reason for collecting less rotated magnetic hysteresis loops at low temperatures. By removing the two contributions, saturated loops were obtained with saturation magnetizations decreasing from 0.98(2) emu/mol at 10 K to 0.75(3) emu/mol at 300 K. While the saturation magnetization at 10 K represents the spontaneous magnetic moments of *N_A_* glass molecules, each glass molecule has to contribute to the spontaneous ferromagnetic signal with a magnetic moment of 1.8 × 10^−4^ µ_B_. However, the ferromagnetic contribution to the magnetization of the Te-5 glass was almost four orders of magnitude lower than of the paramagnetic one, the last one becoming however negligible over the diamagnetic contribution at 300 K mainly in high applied magnetic fields. To note the ferromagnetically coupled magnetic moments can rotate much easier under the magnetic field excitation as compared to the paramagnetic moments and, therefore, under low magnetic fields (e.g., lower than 2,000 Oe) the overall susceptibility was positive and mainly due to the ferromagnetic component. Therefore, of interest for the magnetic response of such a glass under low magnetic fields is the full magnetization reversal process, which was also characterized by the associated coercive field. At a glance of Figure 8b, it can be observed the complex nature of the hysteresis loops at low temperatures, consisting mainly of two components. The most intense one had an impressive coercive field of about 10,000 Oe, decreasing very slightly with the temperature up to 50 K and much faster above 150 K, which has never been reported up to now in any glassy structure.

The second component had a lower contribution and was characterized by a coercive field, which decreased slightly from hundreds of Oe at 10 K to tenths of Oe at 300 K. This is often reported for diluted magnetic oxides [36,37] and even in Te based glasses [38]. To note that an increased coercivity at low temperature can be associated to size dispersed magnetic clusters in the compound, either if they are due to clustering magnetic defects related to oxygen vacancies or to clustering magnetic elements [36]. In the present case, the only sources of interacting magnetic defects are the oxygen vacancies (possible transition metal magnetic impurities can contribute to only the paramagnetic signal) and most probably they are directly related to the formation of the Te_2_ molecules under different clustering degrees. Such metallic-like Te-based colloids may consist of a source of oxygen vacancies around the Te centers, giving rise in turn to nanoclusters of magnetic defects, which can be either isolated (with superparamagnetic behavior at room temperature but of high coercive field at low temperatures) or in magnetic interaction, providing the weak ferromagnetic signal that persists up to room temperature. Given this specific magnetic behavior of the Te-5 glass, the associated magneto-optical behavior will be further investigated.

The Faraday angle (rotation of the polarization plane under an applied field of induction B) was calculated using Equation (8) [9]:

(8) $$\theta_{F}=(\psi_{B+}-\psi_{B=0})$$

where, $\psi_{B+}$ is the ellipsometric angle measured with the applied magnetic field (+0.2 T) and $\psi_{B=0}$ is the ellipsometric angle without an applied magnetic field. Faraday ellipticity would be the difference between ellipsometric parameters Δ (the phase difference), recorded with and without the magnetic field. One should note that no difference between the recorded Δ*(B+)* and Δ*(B=0)* was observed within the equipment and measurement (probing with linear polarized light) accuracy, inferring that the Faraday ellipticity was absent or negligible. It is worth noticing that in the present case of linearly polarized light (for both incoming and transmitted radiations, Δ = 0°), the ellipsometric parameter ψ was equal with the azimuthal angle, θ, and thus the Faraday rotation was given by Equation (8).

**Figure 9:**
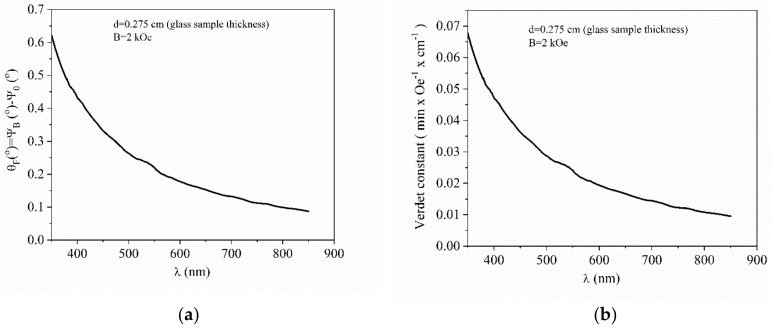
(**a**) Faraday rotation angle vs. wavelength (under *B = 0.2 T*) and (**b**) Verdet constant vs. wavelength for Te-5 glass sample (under *B = 0.2 T*).

In Figure 9a the dispersion of the Faraday rotation angle, *θ_F_,* on the wavelength under the magnetic induction *B* = 0.2 T for the Te-5 glass sample is presented. One should note the decrease of the rotation angle, *θ_F_,* with increasing wavelength, and that the Faraday rotation angle was not affected by the light absorption of the glass in the 450–600 nm range. The reference values were 0.43° at 400 nm and 0.15° at 650 nm.

The Faraday rotation angle, *θ_F_*, is directly proportional with the applied magnetic field, *B*, the geometrical pathway of the light through the glass (e.g., the thickness of the glass), *d*, and a proportional constant, *V*, named Verdet constant that is a characteristic for each material. Verdet constant was calculated using Equation (9) [9,10]:

(9) $$\theta_{F}=VBd$$

Figure 9b presents the wavelength dependence of the Verdet constant as obtained from the Faraday rotation angle values expressed above and via Equation (1) (with *B = 0.2 T* and *d = 0.275 cm*). From Figure 9b a decrease of the Verdet constant with wavelength is noticed, the reference values being 0.047 min·Oe^−1^·cm^−1^ at 400 nm and 0.016 min·Oe^−^·cm^−1^ at 650 nm. Verdet constant values were consistent with the low TeO_2_ content of the investigated glass.

By comparison with alumina-phosphate glasses containing a high amount of Bi_2_O_3_ and PbO, reported by the authors in a previous work related to diamagnetic materials [19], Te-5 glass had twice lower Verdet constant values, measured at 400 nm and 600 nm reference wavelength, due to a reduced amount of TeO_2_ component. It is worth noticing that the Verdet constant of the Te-5 diamagnetic glass was about 5 rad/(T·m) at 650 nm, being one order of magnitude lower than Verdet constant values of currently applied paramagnetic Faraday glasses [39], containing most probably rare-earth ions. These currently applied Faraday glasses were paramagnetic/ferromagnetic materials characterized by a positive susceptibility, usually higher than the negative susceptibility of a diamagnetic material. Hence, the Faraday rotation angle and Verdet constant were higher than those of the diamagnetic Faraday glasses. However, the authors considered that the study of the present phosphate-tellurite glass was justified taking into consideration the low cost of heavy ions-doped glasses compared to rare-earth-doped glasses. Future works will be focused on the investigation of magnetic and magneto-optical properties of high TeO_2_ content glasses as reported in [1].

## 4. Conclusions

A new zinc-phosphate-tellurite glass was prepared by the melting quenching method applying a wet route of processing the precursor reactants. Information on some physicochemical parameters was reported and compared with those of recently investigated tellurite glasses. The optical absorption of the glass was very low at a wavelength higher than 600 nm, which recommends the use of this material as a Faraday rotator. The final product was a composite material comprising of a non-crystalline vitreous phase and Te_2_ nanoclusters, the latter being responsible for the observed specific magnetic behavior. Thermal characteristics revealed a glass network having relatively high energy bonds in the frame of a rigid phosphate-tellurite structure formed by chains and tridimensional units. FTIR and Raman spectroscopy revealed optical phonons specific to the phosphate-tellurite vitreous network. The magnetic measurements show a main diamagnetic behavior of the glass in fields higher than 2000 Oe with superposed paramagnetic contribution at lower temperatures. A weak ferromagnetic phase persisting up to room temperature was also observed, reflected by the complex hysteresis loops due to magnetic centers associated to oxygen vacancies around neutral Te_2_ molecules. Such magnetic defects formed very fine clusters of dispersed sizes, which might be isolated or in interaction. This glass could be a potential candidate for Faraday rotators since, under moderately applied magnetic fields of 1000 Oe, centimeter long glass rods could provide rotation angles of degrees. Improved magneto-optical performances can be expected by increasing the content of Te in the glass. Having in mind the proportionality of the Verdet constant with the magnetic susceptibility and the increase contribution of the paramagnetic phase at lower temperatures, a dependence of the rotational power on the temperature is expected for this material. Hence, it is not only characterized by a reasonable magneto-optical response in low magnetic fields, but can be also used for triggering this functionality by temperature. From this point of view, such an optimized material can be an alternative to the more expensive paramagnetic glasses highly doped by rare-earth magnetic elements.

## Figures and Tables

**Table 1:** Te-5 glass properties: glass density (ρ_glass_), molar volume (V_M_), oxygen packing density (OPD), average molecular mass (M_av_), refractive index measured at 589 nm (n_D_), refractive index measured from dispersion graph (n_DD_), molar refractivity (R_m_), electronic polarizability (α_m_), reflection loss (R_L_) and optical transmission (T).

ρ_glass_ (g/cm^3^)	M_av_ (g/mol)	V_M_ (cm^3^/mol)	OPD O atom/L_glass_	n_D_	n_DD_	R_m_ (cm^3^/mol)	α_m_ (cm^3^/mol)	R_L_	T
2.936	111.45	37.95	75	1.546	1.55	12.02	4.75×10^−24^	0.046	0.912

**Table 2:** Te-5 glass properties: refractive index-based oxide ion polarizability, $\alpha_{O_{2}^{-}}\left(n\right),\;$ band gap-based oxide ion polarizability, $\alpha_{O_{2}^{-}}\left(E_{g}\right),\;$ optical band gap (Eg), refractive index-based metallization criterion, *M(n)*, band gap-based-metallization criterion, *M(E_g_)*, Duffy and Ingram theoretical optical basicity, *Λ_th_*, refractive index-based polarizability-based optical basicity, *Λ*($\alpha_{O_{2}^{-}}\left(n\right)$, band gap-based polarizability-based optical basicity, Λ($\alpha_{O_{2}^{-}}\left(E_{g}\right),$ and Pauling optical basicity, Λ_P_.

$\alpha_{O_{2}^{-}}\left(n\right)\;$ $\left({Å}^{3}\right)$	$\alpha_{O_{2}^{-}}\left(E_{g}\right)$ $\left({Å}^{3}\right)$	*E_g_* (eV)	*M(n)*	*M(E_g_)*	*Λ*_th_	$\Lambda \alpha_{O_{2}^{-}}\left(n\right)$	$\Lambda \alpha_{O_{2}^{-}}\left(E_{g}\right)$	*Λ_P_*
1.59	3.095	3.18	0.683	0.398	0.702	0.62	1.13	0.4617
